# Supplementary figures and images for: Knockout of PA200 improves proteasomal degradation and myelination in a proteotoxic neuropathy
Source: Life Sci Alliance. 2024 Feb 6;7(4):e202302349. doi: 10.26508/lsa.202302349 (PMC10847332; doi:10.26508/lsa.202302349)

|          |   |   |   |   |   |   |   |   |   |   |   |   |
|----------|---|---|---|---|---|---|---|---|---|---|---|---|
| PA200-/- | - | + | - | + | - | + | - | + | - | + | - | + |
| S63del   | - | - | + | + | - | - | + | + | - | - | + | + |

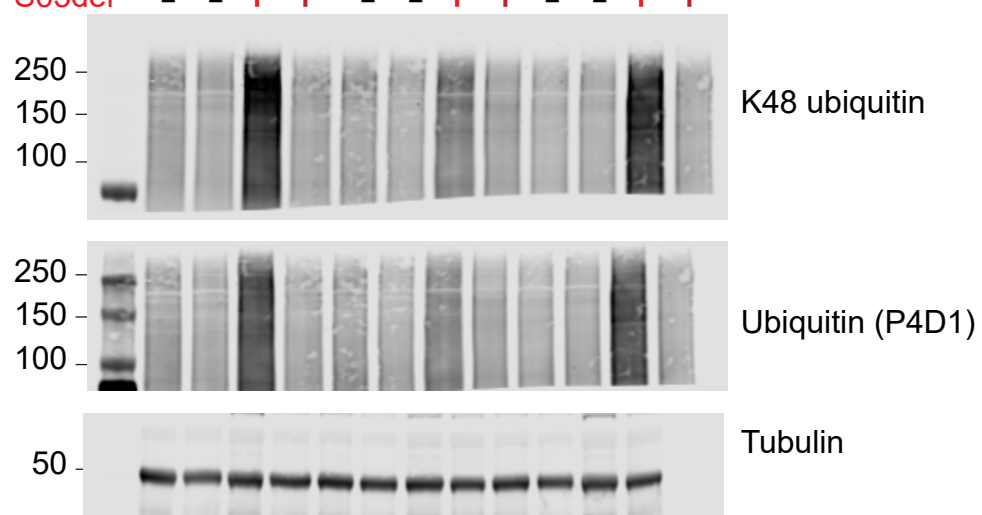

Supplement: Supplementary file 1 [file LSA-2023-02349_SdataF5.pdf]
